# Supplementary material for: Modeling glioblastoma heterogeneity as a dynamic network of cell states
Source: Mol Syst Biol. 2021 Sep 16;17(9):e10105. doi: 10.15252/msb.202010105 (PMC8444284; doi:10.15252/msb.202010105)
Supplement: Supplementary file 5 — Source Data for Figure 3 [file MSB-17-e10105-s001.zip › Figure3A_sourcedata/GSEA_3065/nosigdb_state4.GseaPreranked.1622718430994/gsea_report_for_na_pos_1622718430994.html]

Report for na\_pos 1622718430994 [GSEA]

| GS  follow link to MSigDB | GS DETAILS | SIZE | ES | NES | NOM p-val | FDR q-val | FWER p-val | RANK AT MAX | LEADING EDGE || 1 | LARSSON\_STATE\_6 | Details ... | 101 | 0.99 | 4.20 | 0.000 | 0.000 | 0.000 | 98 | tags=98%, list=2%, signal=98% |
| 2 | NB\_KI\_CLUSTER1 | Details ... | 32 | 0.87 | 2.89 | 0.000 | 0.000 | 0.000 | 711 | tags=94%, list=11%, signal=105% |
| 3 | RICHARDS\_NEFTEL\_CELL\_2019\_AC | Details ... | 34 | 0.70 | 2.40 | 0.000 | 0.000 | 0.000 | 814 | tags=62%, list=13%, signal=71% |
| 4 | SUVA\_AC\_PMID\_31327527 | Details ... | 34 | 0.70 | 2.34 | 0.000 | 0.000 | 0.000 | 814 | tags=62%, list=13%, signal=71% |
| 5 | DOROSZKO\_U3013MG\_CLUSTER\_3 | Details ... | 91 | 0.53 | 2.19 | 0.000 | 0.000 | 0.000 | 1198 | tags=51%, list=19%, signal=62% |
| 6 | RICHARDS\_NEFTEL\_CELL\_2019\_NPC2 | Details ... | 26 | 0.66 | 2.18 | 0.000 | 0.000 | 0.000 | 851 | tags=58%, list=14%, signal=66% |
| 7 | IVY\_IT | Details ... | 27 | 0.67 | 2.16 | 0.000 | 0.000 | 0.001 | 384 | tags=44%, list=6%, signal=47% |
| 8 | ALLEN\_113\_ASTROL1FGFR3FOS | Details ... | 25 | 0.68 | 2.16 | 0.000 | 0.000 | 0.001 | 154 | tags=24%, list=2%, signal=25% |
| 9 | TISIG\_PMID\_32649888 | Details ... | 31 | 0.63 | 2.16 | 0.000 | 0.000 | 0.001 | 1103 | tags=55%, list=18%, signal=66% |
| 10 | SUVA\_NPC2\_PMID\_31327527 | Details ... | 26 | 0.66 | 2.15 | 0.000 | 0.000 | 0.001 | 851 | tags=58%, list=14%, signal=66% |
| 11 | RICHARDS\_ZHONG\_NATURE\_2018\_ASTROCYTES\_UPREG | Details ... | 214 | 0.44 | 2.08 | 0.000 | 0.000 | 0.001 | 1213 | tags=41%, list=19%, signal=49% |
| 12 | GAROFANO\_IAVARONE\_MTC\_SUBTYPE | Details ... | 164 | 0.45 | 2.05 | 0.000 | 0.001 | 0.005 | 1282 | tags=34%, list=20%, signal=41% |
| 13 | OUTERRADIALGLIA | Details ... | 37 | 0.57 | 1.98 | 0.000 | 0.002 | 0.014 | 271 | tags=22%, list=4%, signal=22% |
| 14 | POLLEN\_OUTERRADIALGLIA\_PMID\_26406371 | Details ... | 37 | 0.57 | 1.96 | 0.000 | 0.002 | 0.015 | 271 | tags=22%, list=4%, signal=22% |
| 15 | NB\_KI\_CLUSTER4 | Details ... | 88 | 0.48 | 1.96 | 0.000 | 0.002 | 0.017 | 815 | tags=38%, list=13%, signal=43% |
| 16 | DOROSZKO\_U3180MG\_CLUSTER\_5 | Details ... | 29 | 0.59 | 1.91 | 0.003 | 0.003 | 0.027 | 341 | tags=38%, list=5%, signal=40% |
| 17 | ALLEN\_112\_ASTROL1FGFR3MT1G | Details ... | 25 | 0.57 | 1.83 | 0.003 | 0.008 | 0.064 | 154 | tags=16%, list=2%, signal=16% |
| 18 | RICHARDS\_NOWAKOWSKI\_SCIENCE\_2017\_ASTROCYTE\_UPREG | Details ... | 432 | 0.36 | 1.80 | 0.000 | 0.009 | 0.078 | 1199 | tags=32%, list=19%, signal=37% |
| 19 | RICHARDS\_NOWAKOWSKI\_SCIENCE\_2017\_NIN2\_UPREG | Details ... | 437 | 0.36 | 1.80 | 0.000 | 0.009 | 0.078 | 756 | tags=24%, list=12%, signal=25% |
| 20 | RICHARDS\_NOWAKOWSKI\_SCIENCE\_2017\_VRG\_UPREG | Details ... | 327 | 0.35 | 1.74 | 0.000 | 0.015 | 0.137 | 1213 | tags=32%, list=19%, signal=38% |
| 21 | RICHARDS\_CAHOY\_JNEUROSCI\_2011\_ASTRO\_MATURE |  | 357 | 0.35 | 1.72 | 0.000 | 0.017 | 0.161 | 1276 | tags=29%, list=20%, signal=34% |
| 22 | NB\_KI\_CLUSTER2 |  | 206 | 0.37 | 1.71 | 0.000 | 0.016 | 0.164 | 1440 | tags=39%, list=23%, signal=49% |
| 23 | RICHARDS\_NOWAKOWSKI\_SCIENCE\_2017\_IPC\_NEN3\_UPREG |  | 189 | 0.37 | 1.70 | 0.000 | 0.019 | 0.195 | 840 | tags=31%, list=13%, signal=35% |
| 24 | GANDAL\_ALCOHOLADDICTION\_UP\_PMID29439242 |  | 106 | 0.40 | 1.69 | 0.000 | 0.019 | 0.204 | 927 | tags=30%, list=15%, signal=35% |
| 25 | RICHARDS\_NOWAKOWSKI\_SCIENCE\_2017\_IN\_CTX\_CGE1\_UPREG |  | 226 | 0.34 | 1.63 | 0.000 | 0.031 | 0.322 | 1244 | tags=32%, list=20%, signal=39% |
| 26 | OLSEN\_SCPLIKE |  | 78 | 0.39 | 1.59 | 0.004 | 0.040 | 0.410 | 901 | tags=37%, list=14%, signal=43% |
| 27 | RICHARDS\_NOWAKOWSKI\_SCIENCE\_2017\_EN\_PFC3\_UPREG |  | 160 | 0.34 | 1.55 | 0.000 | 0.053 | 0.510 | 1065 | tags=33%, list=17%, signal=38% |
| 28 | RICHARDS\_NOWAKOWSKI\_SCIENCE\_2017\_MURAL\_UPREG |  | 457 | 0.30 | 1.53 | 0.000 | 0.059 | 0.557 | 765 | tags=24%, list=12%, signal=25% |
| 29 | DOROSZKO\_U3054MG\_CLUSTER\_5 |  | 76 | 0.38 | 1.53 | 0.008 | 0.057 | 0.557 | 1394 | tags=34%, list=22%, signal=43% |
| 30 | RICHARDS\_NOWAKOWSKI\_SCIENCE\_2017\_MGE\_RG1\_UPREG |  | 259 | 0.32 | 1.53 | 0.000 | 0.055 | 0.558 | 1381 | tags=35%, list=22%, signal=43% |
| 31 | RICHARDS\_NOWAKOWSKI\_SCIENCE\_2017\_CHOROID\_UPREG |  | 453 | 0.30 | 1.52 | 0.000 | 0.057 | 0.573 | 891 | tags=27%, list=14%, signal=29% |
| 32 | GAROFANO\_IAVARONE\_NEU\_SUBTYPE |  | 128 | 0.34 | 1.50 | 0.009 | 0.065 | 0.636 | 1243 | tags=29%, list=20%, signal=35% |
| 33 | RICHARDS\_LIDDELOW\_NATURE\_2017\_A1\_REACTIVE\_ASTROCYTES |  | 16 | 0.53 | 1.47 | 0.051 | 0.076 | 0.706 | 546 | tags=31%, list=9%, signal=34% |
| 34 | RICHARDS\_NOWAKOWSKI\_SCIENCE\_2017\_NEN\_LATE\_UPREG |  | 277 | 0.30 | 1.45 | 0.000 | 0.083 | 0.750 | 696 | tags=19%, list=11%, signal=20% |
| 35 | DOROSZKO\_U3013MG\_CLUSTER\_1 |  | 29 | 0.44 | 1.44 | 0.048 | 0.087 | 0.778 | 1039 | tags=55%, list=17%, signal=66% |
| 36 | RICHARDS\_NOWAKOWSKI\_SCIENCE\_2017\_NIN4\_UPREG |  | 106 | 0.34 | 1.43 | 0.017 | 0.090 | 0.799 | 513 | tags=20%, list=8%, signal=21% |
| 37 | RICHARDS\_NOWAKOWSKI\_SCIENCE\_2017\_IN\_CTX\_MGE1\_UPREG |  | 225 | 0.31 | 1.43 | 0.000 | 0.089 | 0.802 | 1563 | tags=36%, list=25%, signal=46% |
| 38 | SUVA\_NPC1\_PMID\_31327527 |  | 32 | 0.43 | 1.43 | 0.058 | 0.088 | 0.804 | 689 | tags=31%, list=11%, signal=35% |
| 39 | RICHARDS\_ZHONG\_NATURE\_2018\_OPC\_UPREG |  | 67 | 0.36 | 1.42 | 0.025 | 0.090 | 0.826 | 814 | tags=28%, list=13%, signal=32% |
| 40 | SUVA\_OPC\_PMID\_31327527 |  | 34 | 0.41 | 1.42 | 0.068 | 0.088 | 0.826 | 346 | tags=21%, list=6%, signal=22% |
| 41 | RICHARDS\_NOWAKOWSKI\_SCIENCE\_2017\_NIN1\_UPREG |  | 118 | 0.34 | 1.40 | 0.018 | 0.096 | 0.856 | 1289 | tags=31%, list=21%, signal=39% |
| 42 | RICHARDS\_NEFTEL\_CELL\_2019\_NPC1 |  | 32 | 0.43 | 1.40 | 0.064 | 0.095 | 0.862 | 689 | tags=31%, list=11%, signal=35% |
| 43 | RICHARDS\_NOWAKOWSKI\_SCIENCE\_2017\_IN\_CTX\_MGE2\_UPREG |  | 157 | 0.31 | 1.39 | 0.015 | 0.096 | 0.872 | 1139 | tags=29%, list=18%, signal=34% |
| 44 | DOROSZKO\_U3054MG\_CLUSTER\_7 |  | 21 | 0.46 | 1.39 | 0.092 | 0.095 | 0.876 | 1165 | tags=48%, list=19%, signal=58% |
| 45 | RICHARDS\_NEFTEL\_CELL\_2019\_OPC |  | 34 | 0.41 | 1.39 | 0.084 | 0.096 | 0.881 | 346 | tags=21%, list=6%, signal=22% |
| 46 | SEGERMAN\_MULTITHERAPY\_RESISTANCE\_FIG5A\_PMID\_27974212 |  | 21 | 0.44 | 1.35 | 0.099 | 0.116 | 0.932 | 1530 | tags=33%, list=24%, signal=44% |
| 47 | PARADA\_T2\_DN\_26461091 |  | 36 | 0.39 | 1.35 | 0.090 | 0.117 | 0.933 | 2442 | tags=56%, list=39%, signal=91% |
| 48 | NB\_KI\_CLUSTER23 |  | 205 | 0.29 | 1.34 | 0.006 | 0.123 | 0.950 | 754 | tags=21%, list=12%, signal=23% |
| 49 | RICHARDS\_NOWAKOWSKI\_SCIENCE\_2017\_IPC\_NEN1\_UPREG |  | 187 | 0.29 | 1.33 | 0.016 | 0.124 | 0.955 | 767 | tags=20%, list=12%, signal=22% |
| 50 | GANDAL\_SCHIZOPHRENIA\_UP\_PMID29439242 |  | 71 | 0.34 | 1.33 | 0.056 | 0.124 | 0.957 | 722 | tags=25%, list=12%, signal=28% |
| 51 | DOROSZKO\_U3054MG\_CLUSTER\_3 |  | 146 | 0.29 | 1.29 | 0.038 | 0.151 | 0.979 | 1496 | tags=34%, list=24%, signal=44% |
| 52 | GANDAL\_BIPOLAR\_UP\_PMID29439242 |  | 55 | 0.34 | 1.29 | 0.089 | 0.155 | 0.983 | 1592 | tags=45%, list=25%, signal=60% |
| 53 | RICHARDS\_VERHAAK\_CANCERCELL\_2010\_PRONEURAL |  | 92 | 0.31 | 1.28 | 0.057 | 0.156 | 0.986 | 803 | tags=22%, list=13%, signal=25% |
| 54 | RICHARDS\_NOWAKOWSKI\_SCIENCE\_2017\_IPC\_NEN1\_UPREG\_1 |  | 106 | 0.30 | 1.28 | 0.076 | 0.154 | 0.987 | 636 | tags=24%, list=10%, signal=26% |
| 55 | DOROSZKO\_U3054MG\_CLUSTER\_0 |  | 52 | 0.34 | 1.27 | 0.106 | 0.162 | 0.990 | 667 | tags=25%, list=11%, signal=28% |
| 56 | NB\_KI\_CLUSTER18 |  | 62 | 0.32 | 1.24 | 0.121 | 0.185 | 0.994 | 832 | tags=31%, list=13%, signal=35% |
| 57 | RICHARDS\_ZHONG\_NATURE\_2018\_MICROGLIA\_UPREG |  | 151 | 0.28 | 1.24 | 0.033 | 0.188 | 0.994 | 1109 | tags=27%, list=18%, signal=32% |
| 58 | SUVA\_MES2\_PMID\_31327527 |  | 35 | 0.36 | 1.23 | 0.150 | 0.189 | 0.994 | 390 | tags=23%, list=6%, signal=24% |
| 59 | RICHARDS\_NEFTEL\_CELL\_2019\_MES2 |  | 35 | 0.36 | 1.22 | 0.196 | 0.203 | 0.998 | 390 | tags=23%, list=6%, signal=24% |
| 60 | WANG\_CLASSICAL\_PMID\_28697342 |  | 30 | 0.37 | 1.22 | 0.173 | 0.201 | 0.998 | 1160 | tags=33%, list=19%, signal=41% |
| 61 | OLSEN\_MESENCHYMAL |  | 67 | 0.30 | 1.21 | 0.137 | 0.212 | 1.000 | 901 | tags=33%, list=14%, signal=38% |
| 62 | RICHARDS\_VERHAAK\_CANCERCELL\_2010\_NEURAL |  | 39 | 0.34 | 1.20 | 0.167 | 0.213 | 1.000 | 1216 | tags=28%, list=19%, signal=35% |
| 63 | IVY\_LE |  | 19 | 0.41 | 1.20 | 0.230 | 0.214 | 1.000 | 531 | tags=32%, list=8%, signal=34% |
| 64 | NB\_KI\_CLUSTER17 |  | 108 | 0.28 | 1.19 | 0.128 | 0.225 | 1.000 | 857 | tags=27%, list=14%, signal=31% |
| 65 | RICHARDS\_NOWAKOWSKI\_SCIENCE\_2017\_OPC\_UPREG |  | 278 | 0.24 | 1.18 | 0.069 | 0.230 | 1.000 | 1041 | tags=24%, list=17%, signal=28% |
| 66 | RICHARDS\_NOWAKOWSKI\_SCIENCE\_2017\_NEN\_EARLY2\_UPREG |  | 277 | 0.24 | 1.17 | 0.062 | 0.234 | 1.000 | 1534 | tags=29%, list=24%, signal=37% |
| 67 | GANDAL\_AUTISM\_UP\_PMID29439242 |  | 64 | 0.30 | 1.15 | 0.180 | 0.264 | 1.000 | 546 | tags=20%, list=9%, signal=22% |
| 68 | DOROSZKO\_U3054MG\_CLUSTER\_10 |  | 180 | 0.25 | 1.13 | 0.119 | 0.281 | 1.000 | 1347 | tags=29%, list=22%, signal=36% |
| 69 | NB\_KI\_CLUSTER13 |  | 81 | 0.27 | 1.13 | 0.189 | 0.280 | 1.000 | 579 | tags=17%, list=9%, signal=19% |
| 70 | GANDAL\_IBD\_UP\_PMID29439242 |  | 43 | 0.31 | 1.13 | 0.260 | 0.278 | 1.000 | 929 | tags=33%, list=15%, signal=38% |
| 71 | RICHARDS\_VERHAAK\_CANCERCELL\_2010\_CLASSICAL |  | 96 | 0.27 | 1.12 | 0.195 | 0.287 | 1.000 | 1496 | tags=31%, list=24%, signal=40% |
| 72 | DOROSZKO\_U3180MG\_CLUSTER\_6 |  | 25 | 0.36 | 1.12 | 0.285 | 0.284 | 1.000 | 847 | tags=28%, list=14%, signal=32% |
| 73 | DOROSZKO\_U3054MG\_CLUSTER\_9 |  | 417 | 0.22 | 1.10 | 0.083 | 0.310 | 1.000 | 1507 | tags=29%, list=24%, signal=35% |
| 74 | RICHARDS\_CAHOY\_JNEUROSCI\_2014\_MOG\_POS |  | 442 | 0.22 | 1.07 | 0.162 | 0.357 | 1.000 | 1457 | tags=29%, list=23%, signal=35% |
| 75 | RICHARDS\_NOWAKOWSKI\_SCIENCE\_2017\_NIN5\_UPREG |  | 212 | 0.23 | 1.07 | 0.264 | 0.365 | 1.000 | 859 | tags=16%, list=14%, signal=18% |
| 76 | ALLEN\_111\_ASTROL1\_6FGFR3ETNPPL |  | 20 | 0.36 | 1.06 | 0.373 | 0.365 | 1.000 | 1603 | tags=50%, list=26%, signal=67% |
| 77 | DOROSZKO\_U3054MG\_CLUSTER\_2 |  | 177 | 0.22 | 1.04 | 0.345 | 0.402 | 1.000 | 1328 | tags=31%, list=21%, signal=38% |
| 78 | RICHARDS\_NOWAKOWSKI\_SCIENCE\_2017\_NIN3\_UPREG |  | 99 | 0.25 | 1.04 | 0.356 | 0.414 | 1.000 | 832 | tags=19%, list=13%, signal=22% |
| 79 | DOROSZKO\_PERIVASCULAR\_INVASION |  | 33 | 0.30 | 1.02 | 0.420 | 0.440 | 1.000 | 901 | tags=33%, list=14%, signal=39% |
| 80 | NB\_KI\_CLUSTER11 |  | 43 | 0.28 | 0.99 | 0.444 | 0.499 | 1.000 | 325 | tags=14%, list=5%, signal=15% |
| 81 | RICHARDS\_NOWAKOWSKI\_SCIENCE\_2017\_NEN\_EARLY1\_UPREG |  | 462 | 0.19 | 0.98 | 0.579 | 0.524 | 1.000 | 1771 | tags=33%, list=28%, signal=42% |
| 82 | RICHARDS\_NOWAKOWSKI\_SCIENCE\_2017\_IN\_STR\_UPREG |  | 138 | 0.20 | 0.90 | 0.751 | 0.688 | 1.000 | 825 | tags=14%, list=13%, signal=16% |
| 83 | DOROSZKO\_U3013MG\_CLUSTER\_0 |  | 26 | 0.28 | 0.89 | 0.604 | 0.713 | 1.000 | 373 | tags=19%, list=6%, signal=20% |
| 84 | ALLEN\_116\_OLIGOL4\_6OPALIN |  | 15 | 0.31 | 0.84 | 0.664 | 0.802 | 1.000 | 330 | tags=13%, list=5%, signal=14% |
| 85 | ROSEN\_KRONA\_TI\_UNPUBL |  | 30 | 0.20 | 0.67 | 0.928 | 0.956 | 1.000 | 1357 | tags=27%, list=22%, signal=34% |
Table: Gene sets enriched in phenotype **na **[plain text format]****
